# Optimizing photoactivation of PA‐mCherry for optical pooled CRISPR screens

**DOI:** 10.1002/2211-5463.70249

**Published:** 2026-05-01

**Authors:** Sravasti Mukherjee, Giulia Zanetti, Bram van den Broek, Kees Jalink

**Affiliations:** ^1^ Division of Cell Biology The Netherlands Cancer Institute Amsterdam The Netherlands; ^2^ Swammerdam Institute of Life Sciences University of Amsterdam The Netherlands; ^3^ Van Leeuwenhoek Centre for Advanced Microscopy (LCAM) Amsterdam The Netherlands; ^4^ Bioimaging Facility The Netherlands Cancer Institute Amsterdam The Netherlands

**Keywords:** confocal microscopy, optical pooled screens, PA‐mCherry, photoactivation, photobleaching

## Abstract

Optical pooled CRISPR screens have become an attractive tool for the rapid identification of genes involved in biological processes. In such screens, mixed populations of cells, each with a single gene knocked out, are screened by microscopy for phenotypes of interest. Identified hit cells can then be tagged by photoactivation of a co‐expressed marker, such as PA‐mCherry, and subsequently isolated by FACS to identify the responsible guide RNA by next‐generation sequencing. Photoactivation is typically performed by selective irradiation of cells with UV light, using either a digital mirror device (DMD), an external fixed UV laser, or, conveniently, by using the 405 nm laser line present in most confocal scanning microscopes. In this study, the latter approach is optimized for PA‐mCherry, a bright red phototag used by us and others in optical pooled screens. We find that although normal scanning with intense 405 nm light can rapidly activate PA‐mCherry, it also leads to rapid photobleaching. Instead, much higher cellular brightness is achieved by limiting intensity and pixel dwell time during scanning, as well as by slightly defocusing the laser. These results should help optimize cell tagging for genotype–phenotype mapping in optical pooled screens, as well as for other applications.

## Graphical abstract

Photoactivatable PA‐mCherry finds widespread use to optically tag individual cells. However, confocal 405 nm UV laser‐scanning (normal scan) is much less efficient than widefield UV illumination, limiting the use of PA‐mCherry on confocal instruments. We remedy this limitation by reporting that rapid and repeated confocal scanning with a low‐intensity, defocused laser spot (optimized scan) outperforms widefield photoactivation.

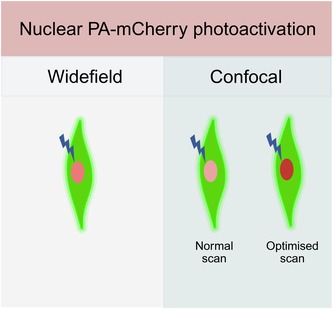

**Abbreviations**
AFC: adaptive focus control
FACS: fluorescence‐activated cell sorting
FLIM: fluorescence lifetime imaging microscopy
FOV: field of view
FRET: förster resonance energy transfer
gRNA: guide RNA
NLS: nuclear localization sequence
OPS: optical pooled screen
PA: photoactivatable
PC: photoconvertible
TCSPC: time‐correlated single photon counting

Photo‐tagging via photoactivatable (PA) and photoconvertible (PC) fluorescent proteins enables selective optical marking of sub‐populations of molecules or cells at defined positions and times, making them indispensable tools for studying living systems. Most commonly, photo‐activation and photo‐conversion are with UV/blue light [1, 2]. At a cellular or tissue level, photo‐tagging enables lineage tracing *in vivo*—marking subsets of cells in developing embryos or regenerating tissues and following their progeny, behavior, and fate [3, 4, 5]. Photo‐tagging also supports quantification of protein turnover, trafficking, and protein exchange between compartments: for example, by converting a defined pool of tagged membrane receptors, their internalization, recycling, or degradation kinetics can be studied in live cells [6].

In recent years, PA‐proteins have also become key to optical pooled CRISPR screens (OPS) [7, 8, 9], where they serve as markers to tag ‘hit cells’, that is, cells in which a gene knockout causes an aberrant phenotype. In a typical OPS workflow (Fig. 1), cells carrying a fluorescent reporter are transduced with a guide RNA (gRNA) library of choice, imaged for a specific phenotype, and analyzed by image‐analysis tools to identify the hit cells and determine their stage coordinates. Then, stage coordinates guide UV photoactivation of hit cells, which are subsequently isolated by Fluorescence‐Activated Cell Sorting (FACS) and genotyped by next‐generation sequencing (NGS) [8, 10, 11]. The success of this approach hinges critically on efficient photo‐tagging of hit cells on the same microscope. Photoactivated cells must be evidently brighter than nonactivated background cells, while neighboring cell activation must be negligible to allow accurate FAC sorting with minimal false positives or loss of hit cells.

**Fig. 1:**
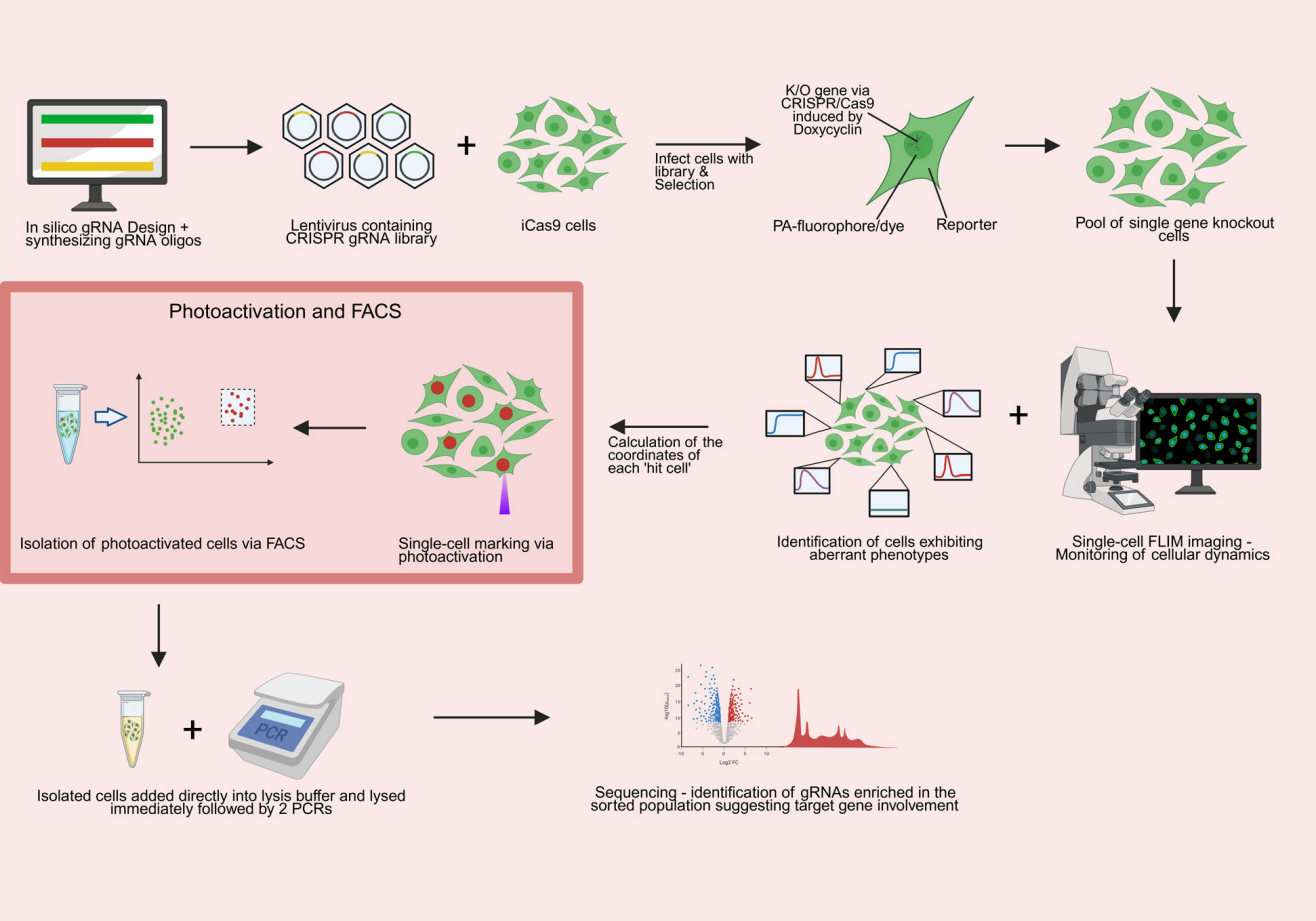
Schematic overview of the optical pooled CRISPR screening pipeline, highlighting the major optimization step (red box). Created in BioRender. Mukherjee, S. https://BioRender.com/zbsl88h.

In this study, we have optimized the scan settings to photoactivate PA‐mCherry for OPS that require a confocal scanning microscope. We initially found that a single confocal scan with a high‐intensity, focused 405 nm diode laser causes photoactivation, but not to the same level attainable with widefield photoactivation, due to significant photobleaching during laser scanning. To overcome this, we developed a novel photoactivation protocol that employs high‐repetition, low‐dose defocused laser scanning to activate PA‐mCherry. Designed to mimic widefield activation in as much as it spreads out the excitation energy over the cell, both in space and in time, we dubbed this method ‘pseudo‐widefield’ scanning. Our method achieves bright, high‐accuracy activation of PA‐mCherry in the cell nucleus, surpassing conventional widefield illumination and thereby enabling rapid cell tagging and high recovery during FACS. Together, these optimizations make PA‐mCherry even better as a versatile and reproducible tool for use not only in screens but also in other confocal imaging workflows.

## Materials

All the materials used in this study are listed in Table 1.

**Table 1:** Materials used in this study.

Reagents	Company/catalog number
Cos7 (green monkey kidney cells)	ATCC, CRL‐1651; Manassas, VA, USA
DMEM + Glutamax	Gibco, ThermoFisher (#61965059); Waltham, MA, USA
Fetal Bovine Serum	Gibco, ThermoFisher (#10270106)
Trypsin	Sigma Aldrich (#T2610); Saint Louis, MO, USA
Trypsin Inhibitor	Sigma Aldrich (#T6522)
Epac‐S^H334^ (Epac‐S^H326^ FRET sensor and 3xNLS‐PA‐mCherry)	Addgene (Plasmid #245738); Watertown, MA, USA
Tol2 transposon system	Kawakami [15]
FuGENE	Promega (#E2311); Madison, WI, USA
G418 (neomycine derivative)	Sigma Aldrich (#G418‐RO)
Penicillin–Streptomycin	ThermoFisher (#15140122)
DMSO	Sigma Aldrich (#D8418)
FluoroBrite medium	Gibco, ThermoFisher (#A1896702)
Polystyrene tube with cell strainer cap	Corning (#352235); Corning, NY, USA
Glass bottom dishes (35 × 10 mm)	Willco Wells (#HBSB‐3522); Amsterdam, The Netherlands
BD FACS Fusion Cell sorter	BD Biosciences; Milpitas, CA, USA

## Methods

### Cell culture

The Cos7 cells (ATCC, CRL‐1651) were cultured in DMEM (+Glutamax) supplemented with 10% FCS. For the creation of the stable cell line, the cell lines were transfected with the Epac‐S^H334^ construct, which contains the Epac‐S^H326^ FRET sensor and 3xNLS‐PA‐mCherry, separated by a self‐cleaving 2A sequence [12, 13, 14]. This construct was transfected using the Tol2 transposon system [13, 15]. Cos7 cells were seeded onto six‐well plates at approximately 10% density and transfected the following day. A mixture of 1 μg of each plasmid with 6 μL of FuGENE reagent per μg of DNA was added to 200 μL of serum‐free DMEM and incubated for 30 min before transfection. Afterward, the mixture was added to the cells for an additional 48 h of incubation. Subsequently, the cells underwent G418 (200 μg/mL) selection to enrich for stably transfected cells, after which the brightest fluorescent population was isolated by FACS sorting. A day before imaging, 150,000–200,000 cells are evenly seeded in glass‐bottom dishes (Table 1).

All cell lines used in this study were authenticated within the last 3 years by short tandem repeat (STR) profiling (Eurofins). In addition, cell morphology and proliferation behavior were routinely assessed to confirm cell line integrity and consistency.

All experiments were performed using mycoplasma‐free cultures. Mycoplasma contamination was routinely monitored using a commercial detection kit (LT07‐318; Lonza, Basel, Switzerland) according to the manufacturer's instructions. Only cultures confirmed to be free of mycoplasma were included in subsequent experiments.

### Imaging and PA‐mCherry activation

Before imaging, cells are removed from DMEM, washed once with PBS, and then 2 mL of FluoroBrite imaging medium is added. The Epac sensor and mCherry were imaged quantitatively using standardized laser settings, using photon counting detectors on a Stellaris‐8 confocal microscope running LAS‐X v4.9.0 software. Full microscope settings are stored with each image as metadata in the .lif files provided in the data repository. In brief, Epac‐S^H334^ was excited at 488 nm (with notch filter) and emission collected at 500–550 nm, and mCherry was excited at 587 nm (notch filter) and emission collected at 629–698 nm in all experiments. For widefield photoactivation, cells in the selected field of view (FOV) were activated by switching on the EL6000 light source of the Stellaris, using a LED 405 nm filtercube, output 213 mW, for 2/5/10/40/60 s. Confocal photoactivation was with the built‐in 405 nm diode laser; see Table 2.

**Table 2:** Photoactivation and readout of PA‐mCherry.

Settings	3xNLS‐PA‐mCherry – readout	3xNLS‐PA‐mCherry – photoactivation
Laser and Intensity	587 nm at 30%	405 nm (internal laser) at 100%
Lens type	20× dry objective; NA = 0.75	20× dry objective; NA = 0.75
Scan mode	512 × 512; Bidirectional	16 × 16; Bidirectional
Resonance scan	8000 Hz	8000 Hz
Zoom (FOV; pixelsize)	2 (290 × 290 μm; 0.57 × 0.57 μm)	48 (12.1 × 12.1 μm; 0.76 × 0.76 μm)
Frame accumulation	none	64
Frames	1	6
Defocusing	none	+ 6 μm
AFC used	Yes	Yes
Duration	N.A.	3 s per nucleus

### Final single‐cell activation with 405 nm diode laser and mCherry read‐out settings

The following optimized settings are used for confocal mCherry imaging and single‐cell photoactivation (Table 2).

### Sample preparation for FACS

After photoactivation, samples are trypsinized and prepared for FACS analysis using a BD FACS Fusion Cell Sorter (BD Biosciences). Appropriate controls were used to set the FACS gating: parental negative cells with no PA‐mCherry, unactivated PA‐mCherry cells (negative control), and maximally activated PA‐mCherry cells (positive control). Maximal activation of PA‐mCherry cells was achieved using a bench‐top 395 nm LED (230 mW/cm^2^) for 6 min, which exactly fit on top of each glass‐bottom dish. Strict gating is used to detect mCherry‐positive cells in the samples. For screening, prioritize high purity over yield to avoid false positives from mis‐sorted cells. Cell recovery at FACS is calculated as follows: Number of mCherry^+^ cells detected/Total number of cells activated.

### Data analysis

All imaging data were first analyzed using Fiji [16]. For data visualization and statistics, GraphPad Prism (v10.3.1) was used.

## Tips and tricks

- Researchers aiming to apply this paradigm should spend some time adapting the precise photoactivation settings to their own cells and microscopes. Exact optimal conditions may vary depending on the objective used and the 405 nm laser power available on their instrument. Limiting the laser power to ~ 0.5 mW and defocusing by about 6 μm should produce good results, but it may be possible to achieve similar results even faster with more powerful 405 nm diode lasers.
- Depending on the capabilities of the instrument, the 6 μm offset can be achieved by changing the Z position of the objective or the stage, with an identical outcome.
- As resonant scanning at 16 lines per image was 0.2 s faster per cell, and as it performs at least equal to 32‐line resonant scanning, we recommend it for OPS.
- Avoid overcrowded seeding in glass‐bottom dishes, as that makes cell segmentation and photoactivation less reliable.
- When preparing samples for FACS, use either trypsin inhibitor or serum‐free DMEM to inactivate trypsin, as serum increases cell stickiness and causes cell doublet formation. Always filter cells through strainer‐cap tubes to eliminate clumps.

## Results

### Generating an optimal cell line

**Fig. 2:**
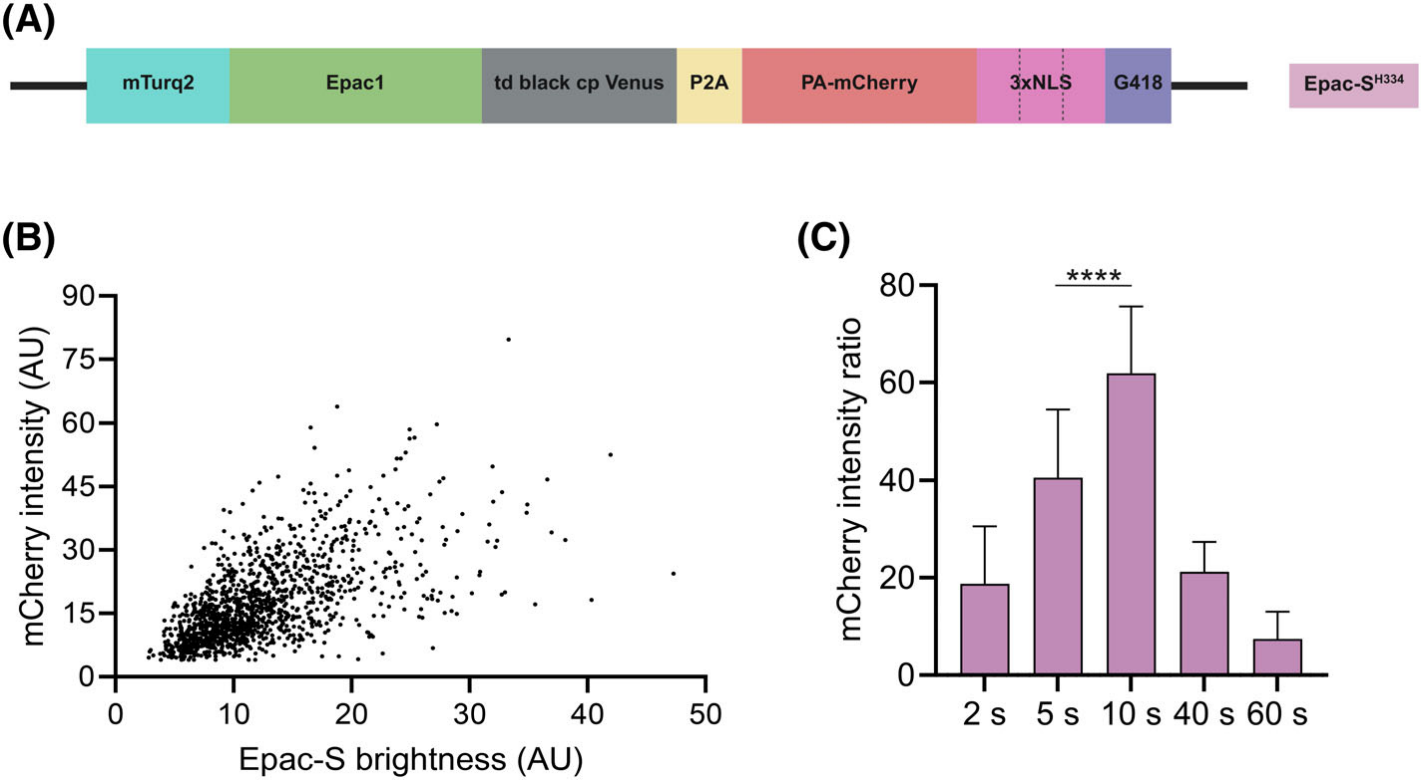
Photoactivation in a Cos7 stable clone coexpressing the Epac sensor and PA‐mCherry. (A) The Epac‐S^H334^ plasmid was used to establish the cell line. (B) Plot of photoactivated PA‐mCherry intensity vs Epac‐sensor intensity shows a strong correlation between expression levels. *R* = 0.60, *P* < 0.001; shown are measured mean intensity per cell. (C) Bar plot showing mCherry intensity ratio (Intensity_post_/ Intensity_pre_) after exposure to widefield 405 nm light for various times; *n* = 20 cells for each condition. 10 s vs 5 s, ****P < 0.0001; statistical analysis was performed using one‐tailed unpaired t‐test. Bars show mean ± SD. (A) was created in BioRender. Mukherjee, S. https://BioRender.com/w6gw6pb

We generated a Cos7 cell line expressing the Epac‐S^H334^ plasmid containing: (a) our high‐affinity dedicated FRET‐FLIM sensor for cAMP, Epac‐S^H326^ [12]; (b) a P2A self‐cleaving peptide; (c) PA‐mCherry, targeted to the nucleus with a 3xNLS sequence; and (d) a G418 selection cassette (Fig. 2A). We chose to limit expression of the phototag to the nucleus, perhaps at the cost of total cell brightness after UV activation, because nuclear expression should help in limiting photoactivation of wild‐type bystander cells. The P2A sequence ensures a 1:1 transcription ratio of the FRET sensor and the PA‐mCherry (Fig. 2B, R = 0.60, *P* < 0.0001), allowing selection of a monoclonal cell line with high mTurquoise and PA‐mCherry expression without first fully photoactivating PA‐mCherry.

### 3xNLS‐PA‐mCherry activation by widefield exposure

We first measured the brightness of the nuclear tag following wide‐field photoactivation using the Leica EL6000 fluorescence lamp at 213 mW for different times (Fig. 2C). Photoactivation was expressed as the ratio Intensity_post photoactivation_/Intensity_pre photoactivation_. Optimal photoactivation, obtained after 10 s, was ~ 60‐fold while significant bleaching was observed at longer exposure times. Thus, we aimed to achieve at least this level of photoactivation with our confocal setup.

### 3xNLS‐PA‐mCherry activation by confocal exposure

**Fig. 3:**
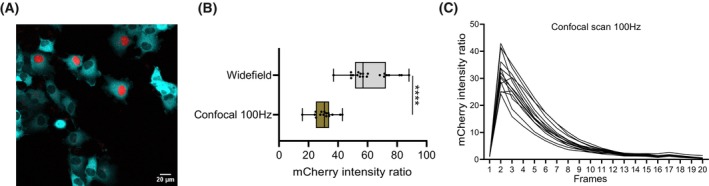
3xNLS‐PA‐mCherry activation by a single 100 Hz confocal scan. (A) selective photoactivation of 4 cells (red nuclei), with minimal neighboring cell activation. The Epac sensor is shown in cyan, mCherry shown in red. (B) mCherry intensity ratio (Intensity_post_/Intensity_pre_) of cells activated with 405 nm widefield vs a single confocal scan at 100 Hz. For Intensity_post_, the frame with the highest intensity after activation was chosen. *n* = 15 cells for each condition. Statistical analysis was by one‐tailed unpaired *t*‐test: ****P < 0.0001. The box shows the median and interquartile range; whiskers indicate the minimum and maximum values; all points are shown. (C) Photoactivation curve showing mCherry intensity ratio change over a time lapse of 20 cycles alternating activation (at 100 Hz) and imaging (at 400 Hz) with confocal scanning; *n* = 15 cells. Note strong bleaching after the first episode. Scale bar, 20 μm.

Next, we tried activating PA‐mCherry with a single confocal scan at 100% power of the 405 nm diode laser, image format 512 × 512 pixels, at 100 lines per second, bidirectional scanning. Additionally, we zoomed in maximally (48×; FOV 12 × 12 μm) so as to hit only a single nucleus and prevent bystander activation. This resulted in robust activation of PA‐mCherry with minimal neighboring cell activation (Fig. 3A), albeit at significantly lower cellular brightness than widefield activation (Fig. 3B). Upon recording a time lapse of 20 alternating imaging/activation cycles, we observed significant photobleaching right from the 2nd cycle (Fig. 3C), likely explaining the lower PA‐mCherry brightness upon confocal scanning at 100 Hz. We therefore set out to identify conditions under which localized single‐nucleus confocal activation is more comparable to widefield activation.

### Mimicking widefield activation while retaining pointing precision

We speculated that intense laser irradiation within the confocal spot may be the cause of high photobleaching, especially since it is hypothesized to increase the population of fluorophores in the triplet state, where fluorophores are much more sensitive to terminal photobleaching [17, 18].

**Fig. 4:**
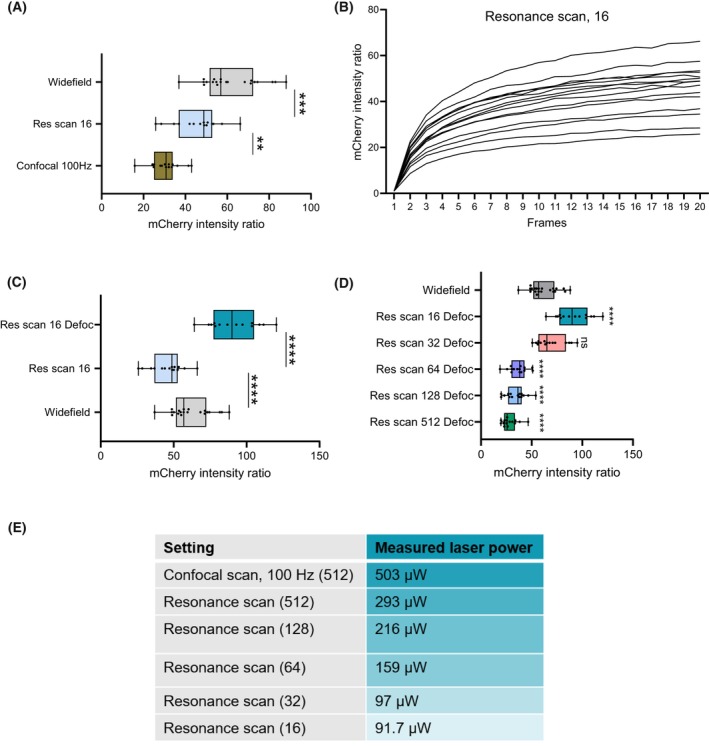
‘Pseudo‐widefield’ activation using resonance scanning and defocusing. (A) mCherry intensity ratio (Intensity_post_/ Intensity_pre_) of cells activated by 405 nm widefield excitation, a single 100 Hz confocal scan at 512 × 512 pixels, and an 8000 Hz line resonance scan at 16 × 16 pixels with 64 frame accumulation. n = 15 cells for each condition. Confocal 100 Hz scan v/s Res 16 scan **P = 0.0014; Res 16 scan v/s Widefield 405 nm ***P = 0.0003. (B) Photoactivation curve of 15 individual cells expressing mCherry, using 16 × 16 format resonance scanning. (C) mCherry intensity ratio of cells activated by widefield 405 nm excitation, by 8000 Hz line resonance scan at 16 × 16 pixels with 64 frame accumulation and by 8000 Hz defocused resonance scan at 16 × 16 pixels with 64 frame accumulation. Widefield v/s Res 16 scan defoc. ****P < 0.0001; Res 16 scan v/s Res 16 scan defoc. ****P < 0.0001. (D) mCherry intensity ratio of cells activated via different resonance scan modes. Resonant scanning at 32 lines was effective (difference with widefield photoactivation: ns, *P* = 0.4898), but at larger image formats resonant scanning became significantly less effective (Difference with widefield photoactivation was significant ****P < 0.0001) for 64, 128, and 512 formats. For A, D, and E, the maximal‐intensity frame after activation was selected. All statistical analyses were performed using a one‐way ANOVA with Tukey's multiple comparisons test. All the boxes in the boxplots show the median and interquartile range; whiskers indicate the minimum and maximum values; all points are shown. *n* = 15 cells for each condition shown from A–D. (E) Measured average laser output power versus scan format on our Stellaris confocal microscope. Note that differences in mean laser powers are caused by differences in laser blanking during line‐ and frame wrap‐around.

In an attempt to deliver as much laser power as possible while limiting the buildup of triplet states, we first limited the time fluorophores are exposed to the intense 405 nm UV focus spot, which relates to the dwell time, by opting for 8000 Hz resonance scanning. For quantification, we compared a 512 × 512, 100 Hz confocal scan to a 16 × 16, resonance scan combined with a 64 frame‐accumulation, both at FOV 12 × 12 μm (zoom 48). Indeed, 16‐line resonance scans led to significantly better photoactivation than 100 Hz scanning (Fig. 4A), most likely by diminished photobleaching as can be deduced from the photoactivation curve (Fig. 4B). However, activation still did not hold up to widefield activation (Fig. 4A; ***P = 0.0003).

To further increase photoactivation efficiency via confocal scanning, we next attempted to reduce bleaching by defocusing by ~ 6 μm, thus avoiding the intense focus point, by setting a positive offset in objective Z position during the photoactivation job. This majorly improved photoactivation and PA‐mCherry cellular brightness, showing even better activation than 405 nm widefield (Fig. 4C).

**Fig. 5:**
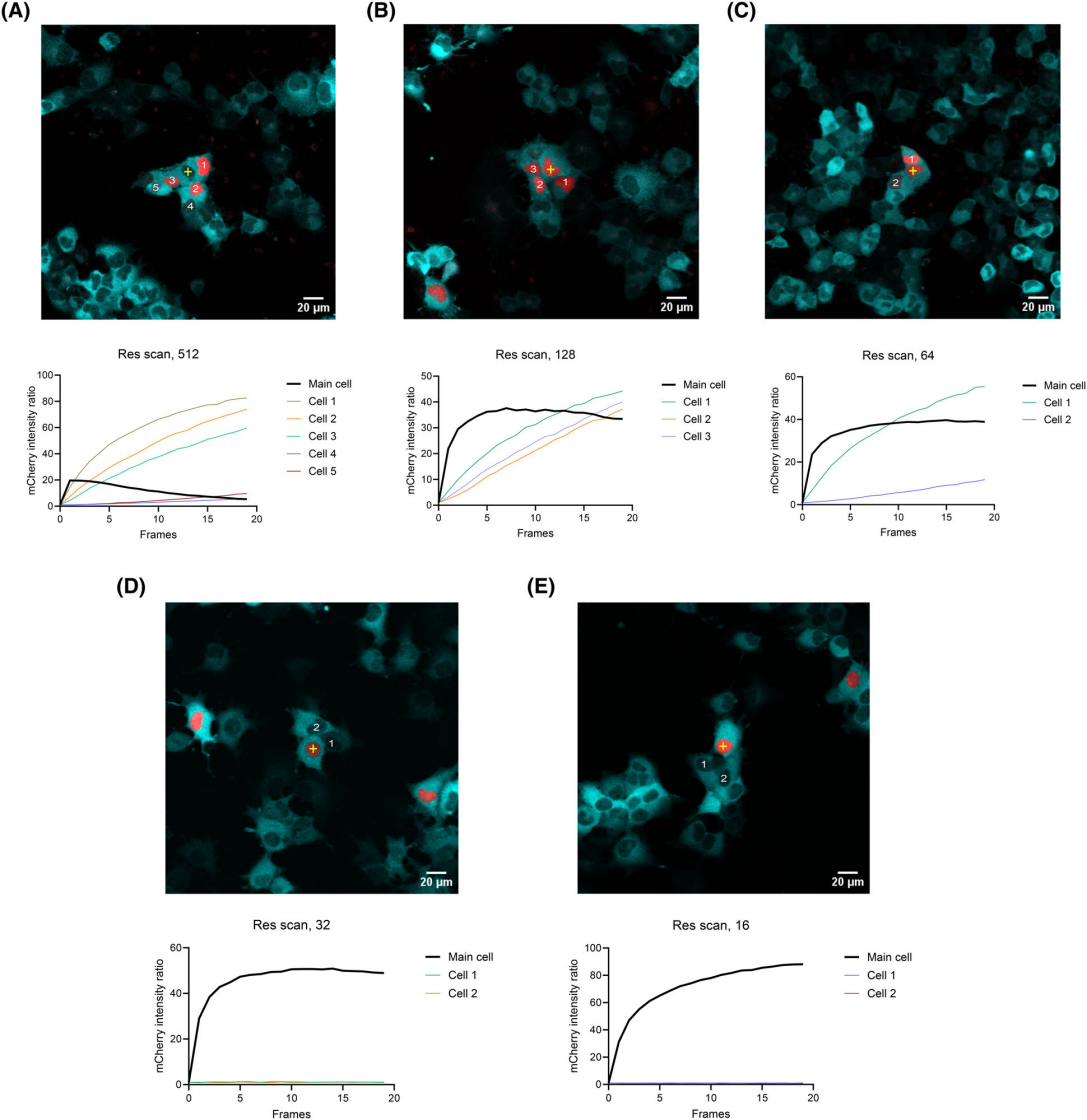
Neighboring cell activation caused by defocused resonance scan modes. (A) 512 resonance scan (B) 128 resonance scan (C) 64 resonance scan (D) 32 resonance scan (E) 16 resonance scan. The image shows the main target cell marked with a yellow ‘+’ and neighboring cells marked by numbers. Graphs show the activation curves in the target cell (black) as well as bystanders, with the corresponding cell number. The shown image is frame 20. Note that depending on the photoactivation regimen, at conditions that cause target cell bleaching, bystander activation can eventually exceed target cell activation. Scale bar, 20 μm.

Encouraged by these results, we also tested photoactivation by defocused resonance scanning at other scan formats (512, 128, 64, and 32) (Fig. 4D). Noting that average laser output varies significantly with different scan formats (Fig. 4E) due to interline and interframe laser blanking, we observed that resonance scan 16 remained the best setting for maximal activation with minimal bleaching. Additionally, we quantified bystander activation in each setting, as defocusing might lead to widefield‐like neighboring cell activation (Fig. 5A–E).

### Optimal PA‐mCherry activation settings for optical pooled screening

**Fig. 6:**
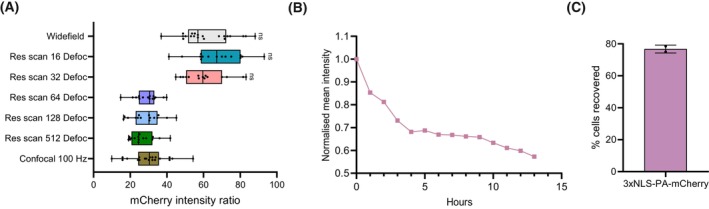
PA‐mCherry photoactivation for pooled screening purposes. (A) mCherry intensity ratio of cells activated for 3 s (i.e., 1 Frame‐Confocal 100 Hz; 1 Frame‐Res scan 512 Defoc; 2 Frames‐Res scan 128 Defoc; 3 Frames‐Res scan 64 Defoc; 4 Frames‐Res scan 32 Defoc; 6 Frames‐Res scan 16 Defoc) at the indicated scan settings. *n* = 15 cells for each condition. Widefield 405 nm v/s Res 16 scan defocus; ns, *P* = 0.7569, Widefield 405 nm v/s Res 32 scan defocus; ns, *P* > 0.9999, Res 16 scan defocus v/s Res 32 scan defocus; ns, *P* = 0.6490. Statistical analysis was by one‐way ANOVA with Tukey's multiple comparisons test. Boxplots show the median and interquartile range; whiskers indicate the minimum and maximum values; each dot is a single cell. (B) Photoconverted signal stability of PA‐mCherry over 14 h post widefield 405 nm photoactivation. (C) Recovery of photoactivated cells by FACS, calculated as: Number of mCherry^+^ cells detected/ Total number of cells activated; *n* = 2 experiments where 200 total cells were activated in each experiment with the optimized settings and subjected to FAC sorting. Bar shows mean ± SD.

In the preceding paragraphs, we have quantified maximal PA‐mCherry activation occurring within the time‐lapse of 20 activation cycles. For OPS, when potentially hundreds or thousands of hit cells must be tagged in a short time span, time limitations need to be considered. Restricting the photoactivation to maximally 3 s per cell, we reanalyzed our data. Under these conditions, defocused resonance scanning at 16 × 16 or 32 × 32 still performed at least equal to widefield excitation. In contrast, larger format resonance scans showed no advantage as compared to 100 Hz line scans. Resonant scanning at 16 lines per image appeared 0.2 s faster per cell, and since it performs at least equal to 32‐line resonant scanning it is recommended for OPS (Fig. 6A).

Finally, we also checked retention of photoconverted 3xNLS PA‐mCherry. Nuclei in living and dividing cells retained at least 50% of their initial brightness for 14 h (Fig. 6B), making it ideally suited for long time‐lapse experiments. Additionally, FACS analysis demonstrated ~ 75–80% cell recovery after photoactivation (Fig. 6C). A 25% loss in FACS is commonly observed due to rejection of doublets and purity‐yield settings.

## Discussion

It has long been recognized that the intense laser radiation encountered in the focus spot of confocal laser scanning microscopes (in this study, 0.4 mW, or > 100 kW/cm^2^ in the focus spot) causes excessive bleaching and low fluorophore photon yield. Such high excitation fluxes quickly drive a substantial fraction of molecules into the triplet ground state (T_0_), where subsequent excitation to an excited triplet state (T_1_) renders them highly reactive and prone to bleaching via redox reactions [17, 18]. Avoiding excited triplet states, either by reducing the laser intensity (as in scanning disk confocal microscopes) or by spreading out the excitation power (as in widefield deconvolution microscopes), prevents excessive triplet‐state buildup and is therefore much milder for both fluorophores and cells. We here used the strategy of limiting the pixel dwell time by low‐resolution resonance scanning [17], combined with defocusing of the laser spot to ~ 6 μm. Our data show that using these conditions, we obtain photoactivation of PA‐mCherry to levels comparable to or exceeding those of widefield photoconversion, despite using less laser power (Fig. 4E), aiding efficient isolation by FACS.

The photobleaching process is still incompletely understood and may involve several molecular states, including triplet states and other dark states, and it is strongly dependent on oxygen levels, which are hard to determine in the nucleus of cells. We therefore did not attempt to model or quantitatively understand what photophysical processes exactly contribute to the excellent outcome of our resonant and defocussed PA‐mCherry activation paradigm.

In our experiments, we aimed at determining conditions for (a) optimal photoconversion, that is, to render the cells as bright as possible so as to optimize their contrast, and (b) to quickly photoactivate PA‐mCherry in a limited timespan (3 s). The latter is of special importance in large pooled screens, when hundreds or thousands of identified live hit cells need to be tagged before the cells have time to migrate noticeably. Zoom was fixed at 48× (FOV 12 × 12 μm) to avoid activating nearby nonhit cells. For both applications, with our 20×, 0.75 NA dry objective, we found optimal results using 16 × 16‐pixel format resonance scanning, focused 6 μm below the cytoplasm.

We note that the mCherry Intensity Ratio reported here is a crude measure: it is determined by dividing nuclear brightness post‐ and prephotoactivation. These signals include small contributions of cell autofluorescence and, thus, are sensitive to the relative contributions of autofluorescence and PA‐mCherry and, thereby, to the expression levels of PA‐mCherry.

Finally, note that our work presents a quick optimization of PA‐mCherry photoactivation for studies that require a confocal instrument, such as our FLIM studies [10] on the Leica Stellaris fast Time Correlated Single Photon Counting (TCSPC) FLIM setup. A systematic comparison of all possible combinations of parameters (laser power, scan format, scan speed, accumulation, etc.) is outside the scope of this work, as the outcome of such a comparison would be highly dependent on actual hardware and laser power available (see Tips and Tricks). We also did not include other hardware options for photoactivation. Digital Mirror Devices and powerful UV lasers may be added to achieve faster single‐cell photoactivation [8] but such solutions add significantly to costs and complexity and are not readily available in most laboratories.

In summary, we describe a convenient ‘pseudo‐widefield’ paradigm to activate PA‐mCherry in single cells with high pointing accuracy, low bystander activation, and achievable on commonly available confocal point scanning microscopes.

## Conflicts of interest

The authors declare no conflicts of interest.

## Author contributions

KJ and SM were involved in conception of the study, design and execution of experiments, and preparation of the manuscript. GZ was involved in bulk cell activation optimizations. SM was involved in optimization of 3xNLS‐PA‐mCherry. GZ was involved in preparation of molecular constructs, construction of stable cell lines. SM, BvdB, and KJ were involved in data analysis, statistics, and preparation of figures. All authors provided critical input during finalization of the manuscript.

## Data Availability

All data sets are available on Zenodo – https://doi.org/10.5281/zenodo.17912829.
